# Efficacy and safety of atezolizumab/bevacizumab in patients with HCC after prior systemic therapy: A global, observational study

**DOI:** 10.1097/HC9.0000000000000302

**Published:** 2023-10-27

**Authors:** Vincent Joerg, Bernhard Scheiner, Antonio D´Alessio, Claudia A.M. Fulgenzi, Martin Schönlein, Lorenz Kocheise, Ansgar W. Lohse, Samuel Huber, Henning Wege, Ahmed Kaseb, Yinghong Wang, Antony Mathew, Andrew Kuang, Mahvish Muzaffar, Yehia I. Abugabal, Shadi Chamseddine, Samuel Phen, Jaekyung Cheon, Pei-Chang Lee, Lorenz Balcar, Anja Krall, Celina Ang, Linda Wu, Anwaar Saeed, Yi-Hsiang Huang, Bertram Bengsch, Lorenza Rimassa, Arndt Weinmann, Rudolf Stauber, James Korolewicz, Matthias Pinter, Amit G. Singal, Hong Jae Chon, David J. Pinato, Kornelius Schulze, Johann von Felden

**Affiliations:** 1 I. Department of Medicine, University Medical Center Hamburg-Eppendorf, Hamburg, Germany; 2 Department of Surgery & Cancer, Imperial College London, UK; 3 Department of Internal Medicine III, Division of Gastroenterology and Hepatology, Medical University of Vienna, Austria; 4 Division of Medical Oncology, Policlinico Universitario Campus Bio-Medico, Rome, Italy; 5 Department of Oncology, Hematology and Bone Marrow Transplantation with Section of Pneumology, University Medical Center Hamburg-Eppendorf, Hamburg, Germany; 6 Department of Gastrointestinal Medical Oncology, MD Anderson Cancer Center, University of Texas, Houston, Texas, USA; 7 Department of Gastroenterology, Hepatology & Nutrition, MD Anderson Cancer Center, University of Texas, Houston, Texas, USA; 8 Department of Internal Medicine, University of Texas Health Science Center, Houston, Texas, USA; 9 Department of Internal Medicine, Baylor College of Medicine, Houston, Texas, USA; 10 Division of Hematology and Oncology, East Carolina University, Greenville, North Carolina, USA; 11 Department of Internal Medicine, Southwestern Medical Center, University of Texas, USA; 12 Department of Internal Medicine, Medical Oncology, CHA Bundang Medical Center, CHA University, Seongnam, Republic of Korea; 13 Department of Medicine, Division of Gastroenterology and Hepatology, Taipei Veterans General Hospital, Taipei; 14 Department of Internal Medicine, Division of Gastroenterology and Hepatology, Medical University of Graz, Austria; 15 Department of Medicine, Division of Hematology/Oncology, Tisch Cancer Institute, Mount Sinai Hospital, New York, New York, USA; 16 Department of Medicine, Division of Hematology/Oncology, University of Pittsburgh (UPMC), Pittsburgh, Pennsylvania, USA; 17 Institute of Clinical Medicine, National Yang Ming Chiao Tung University School of Medicine; Division of Gastroenterology and Hepatology, Taipei Veterans General Hospital, Taipei, Taiwan; 18 Department of Medicine II (Gastroenterology, Hepatology, Endocrinology and Infectious Diseases), Freiburg University Medical Center, Faculty of Medicine, University of Freiburg, Freiburg, Germany; 19 Partner Site Freiburg, German Cancer Consortium (DKTK), Heidelberg, Germany; 20 Department of Biomedical Sciences, Humanitas University, Milan, Italy; 21 Medical Oncology and Hematology Unit, Humanitas Cancer Center, IRCCS Humanitas Research Hospital, Milan, Italy; 22 Department of Internal Medicine I, University Medical Centre of the Johannes Gutenberg University, Mainz, Germany; 23 Department of Translational Medicine, University of Piemonte Orientale, Novara, Italy

## Abstract

**Background:**

Since the introduction of the combination treatment of anti-programmed death-ligand 1 antibody atezolizumab and anti-VEGF antibody bevacizumab (AB), median overall survival in HCC has drastically improved. However, evidence on the efficacy and safety of the novel treatment standard in patients with prior exposure to systemic treatment is scarce. The aim of this global, multicenter, observational study was to evaluate the efficacy and safety of AB in patients after previous systemic therapy.

**Methods:**

We screened our global, multicenter, prospectively maintained registry database for patients who received any systemic therapy before AB. The primary end point was overall survival; secondary end points were time-to-progression, progression-free survival, objective response rate, and safety (rate and severity of adverse events).

**Results:**

Among 493 patients who received AB for unresectable HCC, 61 patients received prior systemic therapy and were included in this analysis. The median age of the study population was 66 years, with 91.8% males. Predominant risk factors for HCC were viral hepatitis (59%) and alcohol (23%). Overall survival for AB was 16.2 (95% CI, 14.5–17.9) months, time-to-progression and progression-free survival were 4.1 (95% CI, 1.5–6.6) and 3.1 (95% CI, 1.1–5.1) months, respectively. The objective response rate was 38.2% (7.3% with complete and 30.9% with partial response). Overall survival was not influenced by treatment line (2nd vs. >2nd) or previous systemic treatment modality (tyrosine kinase inhibitors vs. immune checkpoint inhibitors). Treatment-related adverse events of all grades according to Common Terminology Criteria for Adverse Events were documented in 42.6% of patients, with only 13.1% of grade ≥3, including one death.

**Conclusion:**

In this observational study, AB emerges as a safe and efficacious treatment option in patients with HCC previously treated with other systemic therapy.

## Graphical abstract

**Figure:**
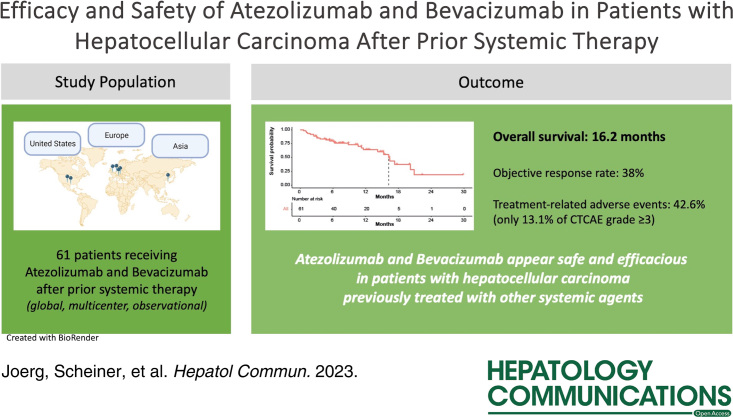

## INTRODUCTION

Despite promising achievements in the treatment landscape of liver cancer,^1^ incidence and mortality are drastically increasing. Predictions estimate 1.4 million new cases and 1.3 million deaths in 2040, which represents an increase in mortality by 56% compared with 2020.^2^

The combination of anti-programmed death-ligand 1 antibody atezolizumab and anti-VEGF antibody bevacizumab has revolutionized systemic therapy for unresectable HCC, which represents the majority of primary liver cancer. Based on the IMbrave150 phase 3 clinical trial, this regimen has become the novel standard of care for first-line systemic therapy,^3,4^ having been proven superior against the multi-tyrosine kinase inhibitor (TKI) sorafenib with an unprecedented median overall survival (OS) of 19.2 months and an objective response rate around 30%.

However, eligibility for the clinical trial was limited to patients without prior exposure to systemic therapy, and therefore, data regarding efficacy and safety in patients who previously received any systemic therapy remain limited. To date, only regorafenib,^5^ cabozantinib,^6^ and ramucirumab (for patients with AFP ≥ 400 ng/mL)^7^ demonstrated a survival advantage against placebo in phase 3 clinical trials enrolling patients with previous systemic therapy (mainly sorafenib).

Overall survival of these regimens ranged between 8.5 and 10.6 months, with a high prevalence of treatment-related adverse events for TKI therapy.^5–8^ Experience of immunotherapy in the second line mostly includes non-randomized, noncontrolled, or early-phase clinical studies^9–13^ There are only 2 phase 3 clinical trials evaluating monotherapy with pembrolizumab against programmed cell death protein 1. However, they reported conflicting results. Early evidence from the KEYNOTE-394 study limited to an Asian population reported a survival benefit for pembrolizumab against placebo, while the global KEYNOTE-240 study failed to demonstrate the superiority of pembrolizumab against placebo.^14,15^

The aim of this global, multicenter, real-world observational study was to assess the efficacy and safety of the combination therapy with atezolizumab and bevacizumab in patients with HCC who previously received other systemic therapies.

## METHODS

### Patient enrollment

We generated a prospectively maintained database, termed atezolizumab bevacizumab (AB real),^16^ including 493 patients who received atezolizumab and bevacizumab for unresectable HCC in 14 tertiary care centers across Europe, the United States, and Asia. The main inclusion criteria were (i) age ≥18 years, (ii) HCC diagnosis according to clinical guidelines,^17^ and (iii) systemic therapy with atezolizumab and bevacizumab. Baseline characteristics and outcomes were provided from a medical chart review by each institution. For this project, patients who were treated with atezolizumab and bevacizumab as first-line systemic therapy were excluded (n = 432), and only patients who previously received any type of systemic therapy were kept as the study population (n = 61).

Clinical baseline characteristics, such as demographic data, etiology, and stage of underlying chronic liver disease or cirrhosis [Child-Turcotte-Pugh score, albumin-bilirubin (ALBI) grade], were obtained by medical chart review and provided by each institution. Performance status as indicated by Eastern Cooperative Oncology Group, tumor stage according to the Barcelona Clinic Liver Cancer (BCLC) staging system, and laboratory tests were recorded at the start of therapy. Information about adverse events (AE) was assessed by local investigators and graded according to the Common Terminology Criteria for Adverse Events.

Patients were followed as per clinical guidelines,^17,18^ which include contrast-enhanced cross-sectional imaging every 2–3 months.

Among our study cohort, 13% (8/61) of patients were previously included in a retrospective analysis of atezolizumab and bevacizumab in HCC with progression after first-line therapy.^12^ This applies to 3 patients contributed from Hamburg and 5 patients from Vienna. Data on the efficacy of atezolizumab and bevacizumab from the remaining 87% (53/61) patients have not been published yet.

### Data analysis

The primary end point of the study was OS from the start of treatment with atezolizumab and bevacizumab. Secondary end points were time-to-progression (TTP), PFS, and investigator-assessed objective response using Response Evaluation Criteria in Solid Tumors 1.1 criteria, as well as safety according to AEs graded by Common Terminology Criteria for Adverse Events v.5.0. For descriptive statistics, continuous variables are reported as median and interquartile ranges and categorical variables as counts and percentages. OS, TTP, and PFS were analyzed using a log-rank test and plotted with Kaplan-Meier curves. The reverse Kaplan-Meier method was used to estimate the median follow-up time. Cox regression modeling was performed for known prognostic clinical variables. A *p*-value below 0.05 was considered statistically significant. All statistical analyses were conducted on SPSS (IBM, version 26) or R studio (R version 4.2.1).

## RESULTS

### Clinical characteristics

**FIGURE 1:**
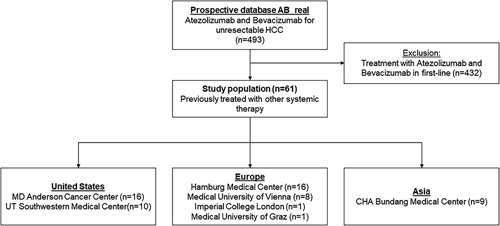
Flow chart of the study with numbers of patients by each contributing center. Abbreviation: AB, antibody bevacizumab.

The overall prospective AB real database currently contains 493 patients from 14 centers globally. For this project, all patients who received previous systemic treatment before atezolizumab and bevacizumab were included in the analysis (n=61) (Figure 1). Baseline characteristics are displayed in Table 1. The median age of the population was 66 (59–71) years, 91.8% of patients were male. Risk factors for HCC were distributed as follows: alcohol in 23%, viral hepatitis in 59%, NASH in 6.6%, and other risk factors in 19.7% of the population. In all, 72.1% of patients had liver cirrhosis. Liver function, according to Child-Pugh Score, was stage A5 in 42.6% (n=26), A6 in 32.8% (n=20), B7 in 14.8% (n=9), B8 in 6.6% (4), B9 in 1.6% (1), and C11 in 1.6% (n=1) of patients. Classification following the ALBI score resulted in 23 patients being classified as grade 1, 34 patients as grade 2, and 4 patients as grade 3. Performance status was mostly adequate, with 98.3% (n=60) of patients being classified as Eastern Cooperative Oncology Group 0 or 1. Tumor stage was assessed according to BCLC staging criteria with 70.5% (n = 43) of patients belonging to BCLC C, 27.9% (n = 17) of patients to BCLC stage B, and 1.6% (n = 1) of patients to BCLC stage A. Extrahepatic tumor extension was present in 44.3% (n = 27) of patients. Median serum AFP levels measured before atezolizumab and bevacizumab exposure were 137 ng/mL IQR 7.1–1.976] and ≥ 400 ng/mL in 39% (n = 23) of patients. With regard to previous systemic treatment, median line of treatment was second line with 63.9% (n=39) of patients having received atezolizumab and bevacizumab as the second line of systemic treatment, 18% (n=11) as third, 6.6% (n=4) as fourth, and 11.4% (n=7) of patients as a further line of treatment, ranging until the seventh line of treatment (n=1, 1.6%). Treatment with atezolizumab and bevacizumab was started after a median of 1 (IQR: 0.4–3.0) month after cessation of the previous regimen. Previous treatment regimens consisted of 1 or multiple TKIs in 47.5% (n = 29), various combinations with immune checkpoint inhibitors (ICI) therapy in 49.2% (n = 30 including n = 5 ICI-monotherapies, n = 3 double ICI-combinations, n = 6 ICI plus anti-VEGF or targeted therapy, n = 13 ICI monotherapy sequentially with TKI treatment, and n = 3 ICI monotherapy with anti-VEGF/targeted therapy or TKI sequentially or combined) as well as 2 patients with prior chemotherapy (3.3%). Overall, 75.4% (n = 46) of patients received at least 1 treatment regimen containing a TKI. The best radiological response to the previous line of systemic treatment was evaluable in 48 (79%) patients. Of these, 1 (2%) patient had a complete response (CR), 6 (10%) had a partial response, 19 (31%) had stable disease, and 22 (36%) had progressive disease as their best radiological response. The most common reason for not starting atezolizumab and bevacizumab as a first-line systemic treatment was lack of approval at the time of systemic treatment initiation (n = 36, 59%), while another 31% of patients (n = 19) decided to participate in clinical trials testing other agents for first line. In 2 patients (3%), atezolizumab and bevacizumab were initially not started at first line due to safety concerns (1 patient with a history of dermatomyositis and fear of autoimmune reactivation, another one with concomitant radiotherapy). In 4 (7%) patients, the reason for not starting atezolizumab and bevacizumab upfront was not well documented.

**TABLE 1:** Baseline characteristics

	Study cohort (n = 61)
Age	66 (59–71)
Sex, n (%)	
Female	5 (8.2)
Male	56 (91.8)
Cirrhosis present	44 (72.1)
Risk factor^b^	
HBV	15 (24.6)
HCV	21 (34.4)
Alcohol	14 (23.0)
NASH	4 (6.6)
Other^a^	12 (19.7)
AFP ng/mL	137 (7–1976)
AFP ≥ 400 ng/ml	23 (39.0)
Child-Pugh Score, n (%)	
A5	26 (42.6)
A6	20 (32.8)
B7	9 (14.8)
B8	4 (6.6)
B9	1 (1.6)
C11	1 (1.6)
ALBI grade, n (%)	
1	23 (37.7)
2	34 (55.7)
3	4 (6.6)
ECOG PS, n (%)	
0	31 (50.8)
1	29 (47.5)
2	1 (1.6)
BCLC stage, n (%)	
A	1 (1.6)
B	17 (27.9)
C	43 (70.5)
Extrahepatic spread	27 (44.3)
Previous nonsystemic treatments^c^, n (%)	
Surgery	23 (37.7)
Ablation	10 (16.4)
TACE	23 (37.7)
TARE	11 (18.0)
EBRT	7 (11.5)
No.of previous systemic treatments, n (%)	
1	39 (63.9)
2	11 (18.0)
3	4 (6.6)
4	2 (3.3)
5	3 (4.9)
6	1 (1.6)
7	1 (1.6)
Median line of systemic treatment	2 (2–3)
Any previous TKI	46 (75.4)
Any previous IO	29 (47.5)

Displayed are medians (IQR) for continuous and frequencies and percentages for categorical variables.

^a^ Includes 2 patients with cryptogenic liver disease, 1 patient with Wilson’s disease, 1 patient with alpha-1-antitrypsin-associated liver disease, 1 patient with a history of liver adenoma that progressed to HCC and 6 patients with unknown etiology of liver disease/no underlying liver disease.

^b^ Patients can have multiple risk factors, i.e. numbers exceed 100%.

^c^ Patients can have multiple previous treatments.

Abbreviations: ALBI, albumin-bilirubin; BCLC, Barcelona Clinic Liver Cancer; EBRT, external beam radiotherapy; ECOG PS, Eastern Cooperative Oncology Group performance status; IO, immune-oncological therapy; TARE/TACE, transarterial radio/chemoembolisation; TKI, tyrosine kinase inhibitors.

Data on upper endoscopy before treatment initiation were available in 48 (79%) patients. Of these, n = 20 (42%) patients had varices at the treatment initiation. While most of these patients (n=19) had esophageal varices, only 4 patients were diagnosed with gastric varices. Overall, 12 patients (60%) had small varices, 7 (35%) patients had medium-sized varices, and one (5%) patient was diagnosed with large varices.

### Efficacy

**FIGURE 2:**
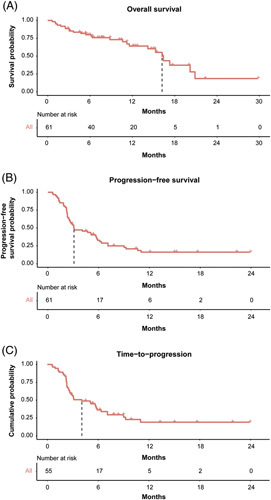
Median overall survival (A), progression-free survival (B), and time-to-progression (C) of the cohort. The latter analysis is limited to patients who had radiologic follow-up data available.

Median OS for the overall cohort was 16.2 (95% CI, 14.5–17.9 Figure 2) months after a median follow-up of 12.2 months (95% CI, 9.5–14.8). 13.1% (n = 8) of patients were still on immunotherapy at the time of data cutoff, and treatment was discontinued in another patient (1.6%) due to ongoing CR. Secondary end points TTP and PFS were 4.1 (95% CI, 1.5–6.6) and 3.1 (95% CI, 1.1–5.1) months, respectively. Median duration of treatment was 3.4 (IQR: 2.0–7.8) months. For evaluable patients (n = 55), the best overall response was assessed as CR in 7.3% (n = 4), partial response in 30.9% (n = 17), stable disease in 27.3% (n=15), and progressive disease in 34.5% (n=19) of patients according to Response Evaluation Criteria in Solid Tumors criteria. For 6 patients (9.8%) no follow-up imaging was available. Disease control rate and objective response rate were 65.5% and 38.2%, respectively.

**FIGURE 3:**
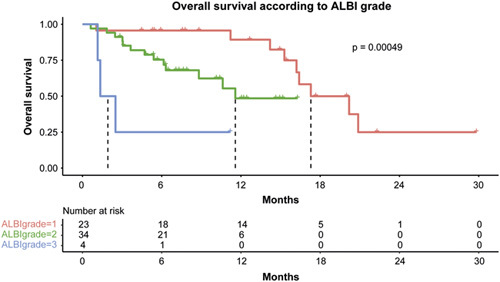
Median overall survival stratified by ALBI grade. Abbreviation: ALBI, albumin-bilirubin.

There was no significant difference in median OS between patients receiving atezolizumab and bevacizumab in the second line (n=38) compared to further lines (n = 23) of treatment (16.4 months 95% CI, 13.5–19.3 vs. 15.3 95% CI, 3.6–26.9, *p* = 0.395). Median OS was 20.2 months (95% CI, 7.1–33.0) in patients without prior exposure to TKI compared to 15.3 months (95% CI, 12.6–18.0) for patients with TKI exposure without reaching significance (*p* = 0.127). In patients with previous immunotherapy, the median OS was 20.2 months (95% CI, 4.6–35.8), whereas the median OS in patients without prior immunotherapy was 16.2 months (95% CI, 14.0–18.4, *p* = 0.874). When stratifying patients by liver function according to ALBI grade, superior liver function was significantly associated with improved OS (*p* < 0.001): ALBI grade 1 17.3 months (95% CI, 12.3–22.3), ALBI grade 2 11.6 months (95% CI, not estimable), and ALBI grade 3 1.3 months (95% CI, 0.027–2.7) (Figure 3). As expected, AFP ≥ 400 ng/ml and ALBI score were independent predictors for worse OS in Cox proportional hazard modeling (HR 3.882, 95% CI, 1.558–9.674, *p*=0.004, and 4.841, 95% CI, 2.242–10.452, *p* < 0.001, respectively) (Table 2).

**TABLE 2:** Cox regression model for death

	Univariable analysis				Multivariable analysis			
		95% CI				95% CI		
	HR	Lower	Upper	*P*	HR	Lower	Upper	*P*
Age	0.979	0.942	1.019	0.300	—	—	—	—
Presence of viral hepatitis	0.956	0.432	2.119	0.913	—	—	—	—
Presence of cirrhosis	1.100	0.455	2.656	0.833	—	—	—	—
AFP ≥ 400	3.103	1.318	7.306	0.010	3.882	1.558	9.674	0.004
ALBI score, per point	3.859	1.953	7.626	<0.001	4.841	2.242	10.452	<0.001
Second vs. later systemic line	1.417	0.633	3.171	0.397	—	—	—	—
BCLC A/B vs. C	0.653	0.277	1.543	0.332	—	—	—	—
Previous IO exposure	1.068	0.477	2.389	0.874	—	—	—	—

Abbreviations: ALBI, albumin-bilirubin; BCLC, Barcelona Clinic for Liver Cancer classification; IO, immune-oncological therapy.

### Safety

Treatment-related AE of any grade were reported in 26 (42.6%) patients during a median treatment duration of 3.4 (IQR: 2.0–7.8) months. Of these, 4 (6.6%) patients experienced a bleeding event (grade 1 in 3 and grade 3 in 1 patient, respectively). Treatment-related AEs of grade 3 or higher were present in 8 (13.1%) patients and included bleeding (n = 1), proteinuria (n = 1) thrombosis (n = 1 leading to death), infections (n = 2), fever (n = 1), gastric ulcer perforation (n = 1), hyperglycemia (n = 1), and osteomyelitis (n = 1) (one patient suffered two AEs). While treatment could be continued in 1 of these patients, it was ultimately stopped in the remaining 7 patients due to concurrent progressive disease (n = 3) or subsequent death (n = 4, 3 of them unrelated to the respective AEs). Furthermore, 4 (6.6%) patients received corticosteroids, and treatment had to be stopped in additional 2 (3.3%) patients due to toxicity of lower grades.

The proportion of patients experiencing AEs of any grade was not associated with the type of treatment modality (TKI or ICI) or ALBI grade. However, AEs were more common in patients treated in the second line as compared with later lines of treatment (second line: n = 20 (52.6%) vs. later lines: n = 6 (26.1%), *p* = 0.042) and numerically higher in patients with CPS class B (64.3%, n = 9/14) and C (100%, n = 1/1) as compared with patients with CPS A (34.8%, n = 16/46; *p* = 0.075).

## DISCUSSION

To our knowledge, we report the first global, prospective, multicenter, observational cohort study on the combination of atezolizumab and bevacizumab for unresectable HCC in patients who were previously treated with at least 1 different systemic therapy regimen. Our data suggest strong efficacy of the combination therapy with a median OS of 16.2 months, objective response rates of 65.5%, and PFS and TTP of 4.1 and 3.1 months, respectively. As expected, the ALBI score was an independent predictor of impaired survival in Cox modeling, and patients stratified as grade 1 according to the ALBI score showed a median OS of 17.3 months. This is comparable to the outcome of patients treated with atezolizumab and bevacizumab in the first systemic line within the IMbrave150 trial^3,4^as well as in real-world studies.^16,19^ As expected, compromised liver function resulted in a decreased median OS (11.6 mo for ALBI grade 2, 1.3 mo for ALBI grade 3), which is also in line with studies from first-line treatment.^20^

Importantly, atezolizumab and bevacizumab treatment was not only effective but also safe, with an AE rate of 42.6% overall and an AE grade ≥ 3 rate of 13.1%. This rate is in line with the IMbrave150 trial^3,4^ and other real-world studies^19^ on first-line treatment with atezolizumab and bevacizumab. In particular, no grade 3 or higher immune-related events occurred, and only 6.6% of patients required corticosteroid treatment, which could be explained by the high number of patients previously tolerating ICI-based therapies. In conclusion, neither the further line setting nor the fact that we included 25% of patients with advanced liver disease (Child-Turcotte-Pugh B/C) seemed to compromise its safety. Comparable safety data have also been reported in other studies evaluating atezolizumab and bevacizumab in patients with advanced liver disease and impaired liver function.^19,21^

It is highly likely that patients progressing to multiple lines of treatment while maintaining preserved liver function and adequate performance status will represent a selected HCC subgroup characterized by a favorable tumor biology. However, this is a potential bias inherent to any studies evaluating second-line treatment regimens, and until now, only limited data were available regarding the efficacy of the combination therapy with atezolizumab and bevacizumab in a further-line setting. Currently, only regorafenib, cabozantinib, and ramucirumab (the latter only for patients with AFP ≥ 400 ng/ml) have yielded positive results in phase 3 clinical trials for patients previously treated with TKI systemic therapy (ie, sorafenib).^1,5–7^ However, the median OS in these studies was only around 10 months (10.6 mo (95% CI, 9.1–12.1), 10.2 months (95% CI, 9.1–12.0), and 8.5 months (95% CI, 7.0–10.6) for regorafenib, cabozantinib, and ramucirumab,^5–7^ respectively). Noteworthy, objective tumor response rates were remarkably higher for the combination therapy compared to other agents, including our study with 38.2%, which is important as a recent meta-analysis including 34 randomized controlled trials in HCC concluded that achieving an objective response is associated with a significantly favorable prognosis.^22^ The radiological responses observed in our study included 7.3% of patients with a CR, and in 1 patient, treatment with atezolizumab and bevacizumab could even be stopped due to an ongoing CR, a pattern of response that has hardly ever been observed in patients treated with TKI.

Systemic treatment sequencing remains challenging in patients with HCC.^23^ Some earlier phase clinical trials have shown promising results for pembrolizumab or nivolumab monotherapy, and nivolumab in combination with ipilimumab in a second-line setting.^11,24,25^ However, while an Asian phase 3 clinical trial evaluating pembrolizumab against placebo in a second-line setting after sorafenib was positive,^14^ the global phase 3 clinical trial did not meet its primary end point.^15^

To our knowledge, there are only 2 studies specifically reporting real-world experience with atezolizumab and bevacizumab after previous systemic therapy, each with a smaller sample size and mostly national cohorts compared to our study. Most recently, a retrospective multicenter study included 12 German and 1 Austrian center and analyzed 50 patients who received atezolizumab and bevacizumab after at least 1 previous line of systemic therapy.^12^ The authors reported a median OS of 16.0 months (95% CI 5.6–26.4), an objective response rate of 32%, and a disease control rate of 68%, almost identical to our results. Only the median PFS was higher compared to our cohort (7.1 mo, 95% CI 4.4–9.8). Notably, data from our study were collected prospectively and included a global cohort of patients from Europe, the United States, and Asia, which further strengthens the generalizability and reproducibility of our results. Another study from Japan limited their analysis to patients who were treated with molecular targeted therapy before receiving atezolizumab and bevacizumab and focused on tumor growth patterns. This study included 31 patients, of whom 20 patients were previously treated with lenvatinib while the remaining 11 patients received other molecular therapies, including sorafenib, regorafenib, and ramucirumab. Patients with prior lenvatinib treatment showed initial tumor growth followed by shrinkage under atezolizumab and bevacizumab, ultimately resulting in higher objective response rates (21% vs. 9%). However, this did not reach statistical significance and median OS was similar between groups as well (11.6 vs. 11.4 mo).^13^

Despite our promising findings regarding the efficacy and safety of atezolizumab and bevacizumab in subsequent treatment lines, our study has several limitations. First, the sample size, although multicenter and global, is rather small, and especially subgroup analyses must be considered exploratory and need to be confirmed in larger studies. Due to the variety of different prior systemic therapies and the limited sample size, subgroup analysis of specific prior treatment regimens was not possible. Secondly, we only included patients who actually received the combination treatment, which could render a selection bias toward patients with favorable tumor biology and other known (eg, age, performance status, and liver function) and unknown prognostic factors. Among our study cohort, 13% (8/61) of patients were previously included in a retrospective analysis of atezolizumab and bevacizumab in HCC with progression after first-line therapy.^12^

In conclusion, our data suggest atezolizumab and bevacizumab as efficacious and safe alternative treatment regimens for patients after previous systemic therapy, including later treatment lines. Nevertheless, larger confirmatory studies are warranted, and the most efficacious sequence of systemic therapy after initial progression is yet to be determined in prospective studies.
